# Endoscopic findings of the stomach in pleasure horses in Poland

**DOI:** 10.1186/1751-0147-55-45

**Published:** 2013-06-07

**Authors:** Artur Niedźwiedź, Krzysztof Kubiak, Józef Nicpoń

**Affiliations:** 1 Department of Internal Diseases with Clinic for Horses, Dogs and Cats; The Faculty of Veterinary Medicine, Wrocław University of Environmental and Life Sciences, pl. Grunwaldzki 47, Wrocław 50-366, Poland

**Keywords:** EGUS, Horse, Gastric ulceration, Prevalence, Pleasure horse, Anatomical distribution

## Abstract

**Background:**

This study was performed to determine the prevalence of ulcers in the gastric squamous and glandular mucosa in Polish pleasure horses.

**Study design:**

Medical records from gastroscopic examinations of 108 pleasure horses of different breeds were reviewed. The study population consisted of two groups; group I (n = 48) with horses that expressed mild clinical signs of gastric ulcer syndrome (EGUS) including poor appetite, slight weight loss or poor body condition, and group II (n = 60) with horses that had no signs of gastrointestinal problems. The age range was 4–10 years, including 5 males, 34 castrated males (geldings) and 69 mares. The prevalence, distribution and severity of gastric ulcers were recorded. Lesions involving the squamous mucosa and the glandular mucosa of the antrum and pylorus were graded and compared between groups.

**Results:**

Significant difference was found in the presence and severity of gastric ulcers between the two groups of horses. The overall prevalence of gastric ulcers in the first group of horses (n = 48) was 59% while in the group of clinically healthy horses (n = 60) the prevalence of gastric lesion was 40% (P = 0.004). Almost 19% of horses from group I had between 6–10 lesions (EGUS score III) and nearly 19% had either >10 localized lesions or very large diffuse lesions (EGUS number score IV). The number of ulcerations in affected horses were significantly lower in group II compared to group I (P = 0.016) as 10% of horses had 6–10 lesions (EGUS number score III) and nearly 14% had either >10 localized lesions or very large diffuse lesions (EGUS number score IV). Gastroscopy revealed that nearly 32% of horses from the second group had an ulceration EGUS score ≥ II.

**Discussion and conclusions:**

This study confirms that gastric ulcerations can be prevalent in apparently clinically normal pleasure horses and a complete gastroscopic examination including the examination of the pylorus is advisable to evaluate this syndrome.

## Introduction

Equine gastric ulcer syndrome (EGUS) is a common health problem in horses and foals and may have an impact on their condition and performance. The diagnosis of EGUS is based on a history of the disease, clinical signs, response to treatment and a gastroscopic examination. The gastroscopic examination has been acknowledged as the “gold standard” of diagnosis because a definitive diagnosis of gastric ulceration in horses can only be achieved by visualizing the lesions in the stomach [1,2].

High intensity training and racing are closely associated with a high prevalence of gastric lesions in both Thoroughbred and Standardbred racehorses. Overuse of nonsteroidal anti-inflammatory drugs (NSAIDs) and corticosteroids, intermittent feeding, and feeding with high-grain and high-calcium diets may also contribute to this condition [3-5]. A study concerning feed management as a possible factor associated with EGUS showed that food deprivation for even 24 hours resulted in a decrease in the median gastric pH to 1.6, compared with a median pH of 3.1 when horses had ad libitum access to hay. High-concentrate diets contain high amounts of digestible carbohydrates, which are enzymatically broken down in the stomach and small intestine and absorbed as glucose and fructose. Generally, there is less buffering from saliva during rapid ingestion of concentrate feeds and acidity in the stomach rises during prolonged intervals between meals. This is believed to be a key factor in the etiology of clinically significant stomach ulcers in animals on low fiber diets [6,7].

Clinical signs of EGUS are numerous and often non-specific. Therefore, not all affected horses show signs of the disease. In race horses, gastric ulceration can be associated with poor performance, acute colic, poor appetite, excessive salivation, chronic diarrhea or skin problems i.e. poor hair coat. The severity of clinical signs may be correlated with gastric lesions, but, in many cases, there is no correlation [8].

The prevalence of the disease in racehorses has been reported to be as high as 100%. In general, it is reported to be approximately 87-90%, in horses that are in race training and 58% in pleasure horses in full work [9-11]. There is little information in literature concerning the prevalence and severity of gastric ulcers in pleasure horses, which are in light or no training.

The purpose of the current study was to analyze the prevalence and distribution of gastric ulceration in a group of Polish pleasure horses in light training.

## Materials and methods

The research was carried out with the approval of the 2nd Local Ethics Committee on Animal Experimentation in Wrocław – resolution No 28/2005 of 14 October 2005. Medical records and endoscopy images of 108 pleasure horses of different breeds that had gastroscopic examinations performed by the authors in an equine clinic during a 5-year period (2006–2011) were reviewed. The study population consisted of two groups; group I (n = 48) with horses that expressed mild clinical signs of gastric ulcer including poor appetite, slight weight loss or poor body condition, and group II (n = 60) with horses that had no signs of gastrointestinal problems. They were examined as a part of a gastroendoscopic survey during classes with students. The total study population included 76 Polish Half Bred Horses, 21 Thoroughbreds, 2 Friesian Horses, 8 Arabian Horses, and 1 Hessisches Warmblood. The age range was 4–10 years, (mean ± standard deviation [SD] 6.9 ± 1.9 years), including 5 males, 34 castrated males (geldings) and 69 mares. Most of the horses were kept in conditions typical for leisure horses in Poland. The horses spent the days on pastures and nights in stables. Mineralized salt blocks and grass hay were supplied during times when pasture conditions were not sufficient for optimal nutrition. Moreover, horses were fed approximately 0.5-1 kg of crushed oat three times daily. Water was provided ad libitum. Horses were also routinely vaccinated against influenza and tetanus and dewormed before, in the middle and after the pasture season. All horses had similar light workloads, which meant they worked lightly five times a week no more than three hours per day and did not participate in competitions for at least three months.

The medical history of the horses was also collected. However, a definitive use of NSAIDs like phenylobutazone or flunixin was stated in only 4 horses.

Water was provided ad libitum and food was withheld for 12–20 hours before endoscopy. Horses were physically restrained in a stock with a nose twitch and 0.02 mg/kg IV of detomidine was administered as needed for sedation. Endoscopic examinations were performed using a 3.25 m videoendoscope (Karl Storz 60332 PKS). The stomach was insufflated with air until its mucosal lining was smooth. Gastric contents were removed from the mucosa by flushing water through the endoscope biopsy channel [12]. The stomach was observed systematically to visualize the entire squamous and glandular mucosa during each gastroscopy. The Number/Severity (N/S) gastric lesion scoring system was used (Table 1) [13,14]. To determine if there was a significant difference between the two examined groups, a Mann–Whitney Rank Sum Test was used. Differences in the prevalence and severity of gastric ulcers between the two groups as well as the effect of age and gender were established using 1-way ANOVA. Statistical analysis was performed using STATISTICA v. 7.0 (StatSoft, Tulsa, OK, USA). A P value of less than 5% (P < 0.05) was considered statistically significant.

**Table 1:** Description of scoring system used

**Lesion number score**	**Description**
0	No lesions
I	1-2 localized lesions
II	3-5 localized lesions
III	6-10 lesions
IV	> 10 lesions or diffuse (or very large lesions)
**Lesion severity score**	**Description**
0	No lesions
I	Lesions appears superficial (only mucosa missing)
II	Small, single, or multifocal erosions or ulcers.
III	Large, single, or multifocal ulcers, or extensive erosions and sloughing
IV	Active hemorrhage or adherent blood clot

## Results

A thorough examination of the squamous nonglandular mucosa was possible in all horses. In the majority of horses a thorough examination of the glandular region was also possible with the exception of a small area where residual fluid was pooling in the ventral aspect of the fundus.

**Figure 1:**
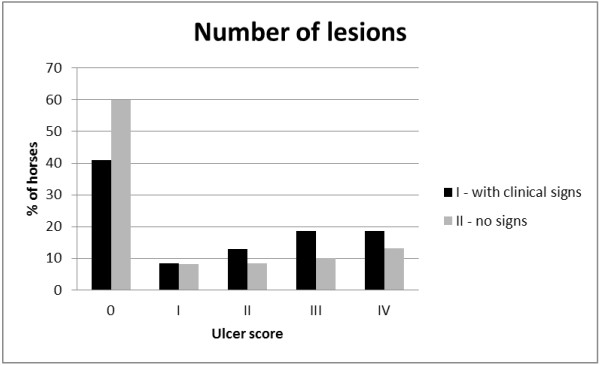
**Lesion number score of gastric ulcers in Polish pleasure horses.** Group I (n = 48): horses that expressed mild clinical signs of gastric ulcer syndrome. Group II (n = 60): horses that had no signs of gastrointestinal problems.

The overall prevalence of gastric ulcers in the group of horses with mild clinical signs was 59% (28/48), and 40% (24/60) in the group of clinically healthy horses (Figure 1). The difference between these two groups was highly significant (P = 0.004). There was no significant difference in the number of lesions between the squamous mucosa and the glandular mucosa of the antrum and pylorus between the two groups (P = 0.89 and 0.75 respectively).

Nearly 19% (9/48) of horses from group I (n = 48) had 6–10 lesions (EGUS number score III) and nearly 19% (9/48) had either >10 localized lesions or very large diffuse lesions (EGUS number score IV). Taking all the diagnosed ulcers into consideration in group I, 50% (24/48) of horses were assessed as having an EGUS score ≥ II. The number of ulcers in affected horses from group II (n = 60) was statistically lower compared to group I (P = 0.016) as 10% of horses (6/60) had 6–10 lesions (EGUS number score III) and nearly 14% (8/60) had either >10 localized lesions or very large diffuse lesions (EGUS number score IV). Gastroscopy revealed that nearly 32% of horses in group II (19/60) had an EGUS ulceration score ≥ II.

In all of the horses, the majority of ulcers were present in the squamous portion of the stomach, near the *margo plicatus*, but the glandular region was also affected to some extent. The severity of lesions in the nonglandular region was statistically different between the two groups (P = 0.012), as in glandular region (P = 0.031). Detailed results are presented in Table 2.

**Table 2:** Location and lesion severity score of gastric ulcers in polish pleasure horses

				**Severity**		
	**Total**	**0**	**I**	**II**	**III**	**IV**
**Group I**	**n = 48**					
*Nonglandular mucosa*		10 (20)	6 (12.5)	10 (20)	16 (35)	6 (12.5)
*Glandular mucosa*		11 (23)	2 (4.1)	10 (20)	22 (45.9)	3 (7)
**Group II**	**n = 60**					
*Nonglandular mucosa*		22 (36)	9 (15)	13 (21.7)	14 (24)	2 (3.3)
*Glandular mucosa*		24 (40)	3 (5)	13 (21.6)	19 (31.8)	1 (1.6)

Group I (n = 48): horses that expressed mild clinical signs of gastric ulcer syndrome. Group II (n = 60): horses that had no signs of gastrointestinal problems (n [%]).

Most young horses aged between 4–6 years (63.7%) had an ulcer score of 0 - II. In the group of horses between 6–10 years-old, most (58.1%) of them had an ulcer score of II-IV. The prevalence of ulcerations was comparable among sex groups.

## Discussion

Gastric ulcerations were present in a large proportion of the examined horses in the two study groups - 59% in group I and 40% in group II. These results are relatively low in comparison with similar studies conducted in sport and show horses, where, depending on the study, the prevalence ranged from 86% [2] to 88.3% [13,15]. A significantly higher prevalence was obtained in this study compared to a population of 3715 horses, older than one year, that were subject to necropsy revealing 7% prevalence [16]. In that study, the occurrence of ulcerations was higher at the lesser curvature (LC) and greater curvature (GC) of the stomach than at the *saccus caecus* (SC), and ulcerations were more severe at the LC than at either the GC or the SC. However, there was no statistically significant association between the presence of lesions in the squamous mucosa and that in the pylorus, which is consistent with findings of others studies [2,8,17].

The main etiological factor of EGUS in pleasure horses seems to be an incorrect diet. The horse’s digestive tract is designed for grazing as the continual feeding, flow of saliva and ingesta buffer the stomach [18]. Intermittent or irregular feeding reduces saliva flow and results in an empty stomach for various periods of time, causing a decrease of gastric pH and exposure of the stomach lining to a more acidic environment. Also, the application of a concentrated diet, which is high in hydrolysable carbohydrates fermentable by resident bacteria results in the production of volatile fatty acids (VFA); which, in the presence of low stomach pH [4], cause damage to the nonglandular squamous mucosa [7]. Grains or other non-fibrous meals are absorbed faster than fibrous meals leading to faster stomach emptying. The constant production of acid when the stomach is empty affects its mucosa [7]. Moreover, grains can produce increased amounts of gastrin, which is one of the main factors for acid production in the stomach [19].

The association between the use of nonsteroidal anti-inflammatory drugs (NSAIDs) and gastric lesions in the examined horses was not critically investigated because of incomplete information on NSAID usage in these horses. NSAID usage within 1 month prior to gastroscopy was confirmed in 4 cases. One horse, which received NSAID, had no visible ulcers and the remaining 3 horses had grade I severity ulcers. This suggests that in this subpopulation, NSAIDs used within reference doses, were not a significant factor in the formation of gastric ulcers which is in an agreement with earlier report [9].

## Conclusions

This study confirms that gastric ulceration can be prevalent in apparently clinically normal pleasure horses and in horses with mild clinical signs of gastric ulcers that are not in intensive work. Furthermore, a complete gastroscopic examination, including an examination of the pylorus, is advisable to evaluate this syndrome. The high prevalence of gastric ulcers in pleasure horses without clinical signs of gastrointestinal disease as described in the present study is a challenge for the clinician, particularly when determining the significance of this finding. One may wonder whether this is a more or less clinically normal or pathological findings in the described group of horses. The obviously multifactorial nature of the equine gastric ulcer syndrome makes prevention difficult. Since the prevalence of gastric ulcers in pleasure horses without clinical signs of disease is so high, gastroscopy should be considered as a diagnostic tool during a periodic health examination. Further studies are needed to determine the etiology of the syndrome and to find ways, if possible, to reduce the frequency of the occurrence of gastric ulcers.

## Competing interests

None of the authors of this paper have a financial or personal relationship with other people or organizations that could inappropriately influence or bias the content of the paper.

## Authors’ contribution

AN designed the study, carried out the endoscopy work. KK performed statistical calculations and coordinated editing and revision of the manuscript. JN coordinated the writing and editing of the manuscript. All authors read and approved the final manuscript.
